# Relation of early-stage renal insufficiency and cardiac structure and function in a large population of asymptomatic Asians: a cross-sectional cohort analysis

**DOI:** 10.3389/fneph.2023.1071900

**Published:** 2023-05-12

**Authors:** Pei-Chen Wu, Kuo-Tzu Sung, Jiun-Lu Lin, Ta-Chuan Hung, Yau-Huei Lai, Cheng-Huang Su, Hung-I. Yeh, Chih-Jen Wu, Chung-Lieh Hung

**Affiliations:** ^1^ Division of Nephrology, Department of Internal Medicine, MacKay Memorial Hospital, Taipei, Taiwan; ^2^ Institute of Biomedical Sciences, Department of Medicine, MacKay Medical College, New Taipei, Taiwan; ^3^ Institute of Biomedical Sciences, Division of Cardiology, Department of Internal Medicine, MacKay Memorial Hospital, Taipei, Taiwan; ^4^ Division of Endocrinology and Metabolism, Department of Internal Medicine, MacKay Memorial Hospital, Taipei, Taiwan; ^5^ Mackay Medicine, Nursing and Management College, Taipei, Taiwan; ^6^ Graduate Institute of Medical Sciences and Department of Pharmacology, School of Medicine, College of Medicine, Taipei Medical University, Taipei, Taiwan; ^7^ Department of Medical Research, China Medical University Hospital, China Medical University, Taichung, Taiwan; ^8^ Institute of Biomedical Sciences, Mackay Medical College, New Taipei, Taiwan

**Keywords:** chronic kidney disease, echocardiography, left ventricular diastolic dysfunction, N-terminal pro-brain natriuretic peptide, proteinuria

## Abstract

**Background:**

Few studies have addressed early-stage kidney disease and preclinical cardiac structural and functional abnormalities from a large-scale Asian population. Further, the extent to which measures of myocardial function and whether these associations may vary by testing various formulas of renal insufficiency remains largely unexplored.

**Objective:**

To explore the associations among renal function, proteinuria, and left ventricular (LV) structural and diastolic functional alterations.

**Design:**

A cross-sectional, retrospective cohort study.

**Setting:**

Registered data from a cardiovascular health screening program at MacKay Memorial Hospital from June 2009 to December 2012.

**Participants:**

Asymptomatic individuals.

**Measurements:**

Renal function was evaluated in terms of estimated glomerular filtration rate (eGFR) by both MDRD and CKD-EPI formulas and severity of proteinuria, which were further related to cardiac structure, diastolic function (including LV e’ by tissue Doppler), and circulating N-terminal pro-brain natriuretic peptide (NT-proBNP) level.

**Results:**

Among 4942 participants (65.8% men, mean age 49.4 ± 11.2 years), the mean CKD-EPI/MDRD eGFR was 90.6 ± 15.7 and 88.5 ± 16.9 ml/min/1.73m^2^, respectively. Lower eGFR, estimated either by the MDRD or CKD-EPI method, and higher proteinuria were significantly associated with lower LV e’ and higher NT-proBNP (all p<0.05) even after adjusting for clinical covariates. In general, lower eGFR estimated by CKD-EPI and MDRD displayed similar impacts on worsening e’ and NT-proBNP, rather than E/e’, in multivariate models. Finally, lower LV e’ or higher composite diastolic score, rather than E/e’, demonstrated remarkable interaction with eGFR level estimated by either CKD-EPI or MDRD on circulating NT-proBNP level (p _interaction <_0.05).

**Limitations:**

Proteinuria was estimated using a urine dipstick rather than more accurately by the urine protein-to-creatinine ratio. Also, pertaining drug history and clinical hard outcomes were lacking.

**Conclusion:**

Both clinical estimate of renal insufficiency by eGFR or proteinuria, even in a relatively early clinical stage, were tightly linked to impaired cardiac diastolic relaxation and circulating NT-proBNP level. Elevation of NT-proBNP with worsening renal function may be influenced by impaired myocardial relaxation.

## Introduction

Chronic kidney disease (CKD) carries an unambiguous risk for a broad spectrum of cardiovascular diseases (CVD), among which heart failure (HF) remains the most common chronic clinical manifestation in patients with CKD (1, 2). The risk of HF rises in accordance with a decline in glomerular filtration rate (GFR) and is greatest in patients with end-stage renal disease requiring dialysis (3). It has been proposed that advanced CKD is characterized by accelerated atherosclerosis (4) and large arterial remodeling, secondary to pressure or volume overload (5), and possibly indolic uremic toxins (6, 7). These factors, when taken together, may lead to unfavorable cardiac remodeling from reduced arterial compliance, increased pulse pressure, and left ventricular hypertrophy (LVH) or fibrosis closely associated with a stiffened left ventricle and impaired diastolic relaxation (2, 8). As a consequence, based on the Frank–Starling law, an acute elevation of preload can cause increased left atrial pressure and pulmonary edema despite apparently preserved ventricular systolic function (9, 10).

A number of mechanisms illustrate the bidirectional interactions between myocardial dysfunction and kidney disease (11); however, it remains unclear whether this interplay may start to take place at a relatively early, clinically asymptomatic stage. Furthermore, various estimates of GFR have been proposed (e.g., CKD Epidemiology Collaboration [CKD-EPI] (12) and four-variable Modification of Diet in Renal Disease [MDRD] (13) formulas), although their impacts on cardiac structural and functional alterations in earlier stages of renal insufficiency have not been fully explored. On the other hand, assessment of diastolic dysfunction (DD) as precursor of HF (14, 15), albeit its complexity with diversity, can be readily assessed using non-invasive echocardiography (16, 17). However, the extent to what degree these indices may be affected and whether these estimates may be equally influenced by renal insufficiency at an earlier stage remains largely unexplored in large-scale Asian population. Here, we aimed to investigate the association between renal function and echocardiographic measurement of diastolic function in asymptomatic individuals.

## Methods

### Data source and study population

This cross-sectional study included asymptomatic participants in an ongoing cardiovascular health screening program from June 2009 through December 2012 at a tertiary-care teaching institute in Northern Taiwan. The primary aim of this program was to examine the hypothesis that certain demographic characteristics, behavioral factors, or biochemical data are associated with subclinical cardiac dysfunction in otherwise healthy individuals. All participants underwent a thorough evaluation, including general physical examination, baseline anthropometric measurements, blood sampling, and comprehensive echocardiography on the day of appointment. As described in our previous work (18), clinical symptoms, baseline comorbidities, smoking status, and exercise habits were obtained from a detailed structured questionnaire. This study was approved by the institutional review board of MacKay Memorial Hospital (14MMHIS202), and conducted in accordance with the Declaration of Helsinki. Informed consent was obtained from all participants.

Baseline comorbidities collected included diabetes, hypertension, dyslipidemia, and CVD. CVD constituted a group of diseases including coronary artery disease (CAD), stroke, and peripheral arterial disease. Laboratory parameters measured included hemoglobin, fasting blood sugar, lipid profile, renal function, N-terminal pro-brain natriuretic peptide (NT-proBNP), and urinalysis. All biochemical tests were conducted using a Hitachi 7170 Automatic Analyzer (Hitachi Corp., Hitachinaka, Ibaraki, Japan), and NT-proBNP was measured using an electrochemiluminescence immunoassay “ECLIA” assay (Roche Diagnostics GmbH, D-68298, Mannheim, Germany) in a standardized central laboratory. Renal function in terms of estimated glomerular filtration rate (eGFR) was calculated using CKD-EPI and four-variable MDRD equations, and was categorized as 30 to < 60, 60 to < 90, and ≧ 90 ml/min/1.73 m^2^. For simplicity, eGFR is referred to as CKD-EPI eGFR if not otherwise specified. We defined proteinuria, measured with a dipstick, as negative, mild (trace to 1+), or severe (2+ to 3+). Test strips were measured using an automatic dipstick analyzer (CLINITEK Novus^®^, Siemens). Validation of results with quantitative urine albumin amount was good (**Supplemental Figure 1**). As per the Kidney Disease: Improving Global Outcomes (KDIGO) guidelines, participants were further classified based on eGFR and proteinuria categories (19). Subjects with missing data for serum creatinine or dipstick proteinuria were excluded from analysis.

### Echocardiographic evaluation

Conventional echocardiography and TDI were performed on all participants, based on the American Society of Echocardiography and European Association of Cardiovascular Imaging guidelines (20, 21) using a GE system (Vivid i, GE Vingmed Ultrasound, Norway) equipped with a 2- to 4-MHz transducer (3S-RS). LV and left atrial (LA) structural parameters measured included LV end-diastolic and end-systolic diameters, wall thickness, LA/LV volume by modified biplane Simpson’s method, and LV mass by the Devereux formula (22). Maximum LA volume (LAVmax) was measured at ventricular end-systole just before opening of the mitral valve, while minimum LA volume (LAVmin) was measured at end-diastole, just before closure of the mitral valve. LV ejection fraction (LVEF) was calculated as 100 × (maximal LV volume − minimal LV volume)/maximal LV volume. LVEF was considered abnormal if < 50%. LV mass was further indexed to body surface area (BSA) as LV mass index (LVMI), and LAV was similarly indexed to BSA. LVH was defined as an LVMI greater than 115 g/m^2^ in men and 95 g/m^2^ in women (23).

The most important modalities to evaluate diastolic function are transmitral pulsed-wave Doppler flow and tissue Doppler mitral annular velocity profile (16, 17). The former helps to assess the presence and severity of DD, which alters the relationship between peak velocity flow in early diastole (E-wave) and that in late filling (A-wave), the time taken from the maximum E to baseline (deceleration time [DT]), and the interval between closure of the aortic valve and opening of the mitral valve (isovolumetric relaxation time [IVRT]). TDI measures the velocity of mitral annular motion, characterized by peak systolic velocity (s’), early diastolic velocity (e’), and late diastolic velocity (a’) in apical four-chamber view. Average e’ was taken as the average of septal e’ and lateral e’. LV filling pressure was estimated using the E/e’ ratio (average e’). DD was defined as E/e’> 15 or average e’ <9 cm/s when E/e’ is between 8 and 15 (24). Composite diastolic score was calculated based on TDI e’ velocity, E/e’ ratio, LAV index, and pulmonary artery pressure (16). Scores ranged from 0 to 2, where 0 was normal, 2 abnormal, and 1 in-between.

All echocardiographic images were performed blind to clinical information by an experienced technician, and stored digitally and reviewed offline using proprietary software (EchoPAC version 10.8, GE Vingmed Ultrasound, Norway). The reproducibility analysis has been reported in our previous article (18). We randomly selected 50 subjects for coefficient of variation analysis of a number of measured parameters (**Supplemental Table 1**). For instance, the intra-class correlation coefficients for LAVmax were 92% between analyzers (interobserver) and 98.5% for the same analyzer (intraobserver).

### Statistical analysis

This study analyzed the relationship between degree of renal dysfunction and cardiac deformational functional changes. The cohort was divided into eGFR and proteinuria categories. Trend tests were performed for continuous variables across categories of eGFR and proteinuria using one-way analysis of variance (ANOVA) and for categorical ones using the Cochran–Armitage test. Continuous variables are presented as mean ± standard deviation (SD); discrete variables are described as counts and percentages.

Multivariate linear regression was performed for markers of DD and renal function. Model 1 was adjusted for baseline clinical features (age and gender). Model 2 was additionally adjusted for baseline comorbidities (hypertension, diabetes, and CVD), body mass index (BMI), systolic blood pressure (SBP), current smoking, and laboratory data (fasting glucose, high-density lipoprotein [HDL], and total cholesterol). Model 3 added proteinuria to model 2. As for sensitivity tests, key echocardiographic variables (LVMI, LVEF, and stroke volume [SV]) were separately added to models 2 and 3. The final results of multivariate analyses were summarized by β-coefficient and 95% confidence intervals (CI).

Because NT-proBNP is a powerful indicator of HF (25), we also tested whether associations between renal function and diastolic parameters vary with NT-proBNP as an *a priori* hypothesis; therefore, possible interactions was evaluated with or without interaction terms between renal function (i.e., eGFR and proteinuria categories) and diastolic parameters (i.e., average e’, composite diastolic score, and LAV index) with NT-proBNP in factorial (two-way ANOVA in SPSS) and linear (ggplot2 package in R) designs.

All statistical analyses were carried out using Microsoft Excel 2013, IBM SPSS Statistics for Windows, Version 22.0 (IBM Corp. Released 2013. Armonk, NY), and R (R Core Team (2022). A two-sided *p*-value <0.05 was considered significant.

### Role of the funding source

No funding was used for this study.

## Results

### Baseline demographics

Our study included 5526 asymptomatic participants, and 584 were excluded for lack of serum creatinine or urine dipstick test (**Supplemental Tables 2**; **3**). Among 4942 enrollees, 65.8% were men, mean age was 49.4 ± 11.2 years, and mean CKD-EPI eGFR was 90.6 ± 15.7 ml/min/1.73 m^2^ at enrollment (**Table 1**). Hypertension was the most prevalent systemic disease in this cohort, reported in 18.7% of the enrollees. All participants were categorized into three groups based on eGFR and into three groups based on proteinuria on a dipstick (**Table 1**). Great heterogeneity was observed between groups in terms of patient characteristics, baseline comorbidities, and laboratory data. As eGFR declined or proteinuria increased, there were trends of greater age, larger BMI, higher blood pressure, higher fasting glucose, higher uric acid, higher triglyceride, and higher NT-proBNP levels (all *p* for trends < 0.05).

**Table 1:** Clinical characteristics of the entire cohort graded by eGFR and proteinuria.

	All (n = 4942)	CKD-EPI eGFR			*p* for trend	Proteinuria on Dipstick			*p* for trend
		≥ 90 (n = 2556)	60-89 (n = 2235)	30-59 (n = 151)		None (n = 3835)	Mild (n = 1030)	Severe (n = 77)	
Patient characteristics									
Age (year)	49.4 ± 11.2	45.3 ± 10.1	52.9 ± 10.4	65.3 ± 10.7	< 0.001	49.1 ± 11.2	49.8 ± 11.1	56.3 ± 12.3	< 0.001
Male gender	3254 (65.8%)	1448 (56.7%)	1696 (75.9%)	110 (72.8%)	< 0.001	2508 (65.4%)	693 (67.3%)	53 (68.8%)	0.21
Height (cm)	165.6 ± 8.5	165.1 ± 8.7	166.4 ± 8.3	164.2 ± 8.2	0.25	165.7 ± 8.6	165.6 ± 8.4	163.5 ± 9.1	0.02
Weight (kg)	67.4 ± 12.9	66.0 ± 13.7	68.8 ± 11.7	69.7 ± 12.4	0.001	67.2 ± 12.5	67.9 ± 14.0	71.5 ± 14.6	0.003
BMI (kg/cm^2^)	24.4 ± 3.6	24.1 ± 3.8	24.7 ± 3.3	25.8 ± 3.8	< 0.001	24.3 ± 3.5	24.6 ± 4.1	26.6 ± 4.5	< 0.001
Body fat (%)	26.2 ± 6.7	26.8 ± 7.0	25.6 ± 6.3	27.0 ± 8.1	0.76	26.2 ± 6.6	26.4 ± 7.0	27.9 ± 8.4	0.03
SBP (mm Hg)	122.9 ± 17.2	120.2 ± 16.6	125.2 ± 16.9	134.9 ± 20.6	< 0.001	122.5 ± 16.5	123.2 ± 18.7	137.7 ± 22.8	< 0.001
DBP (mm Hg)	75.8 ± 10.9	74.4 ± 10.8	77.3 ± 10.7	78.9 ± 12.8	< 0.001	75.6 ± 10.6	76.1 ± 11.5	82.4 ± 13.9	< 0.001
Pulse rate (/min)	74.4 ± 10.2	74.9 ± 10.3	73.7 ± 9.9	76.9 ± 12.2	0.02	74.1 ± 10.0	75.3 ± 10.6	79.5 ± 13.4	< 0.001
Smoking	543 (11.0%)	284 (11.1%)	243 (10.9%)	16 (10.6%)	0.76	389 (10.1%)	144 (14.0%)	10 (13.0%)	0.001
Exercise	704 (14.2%)	353 (13.8%)	333 (14.9%)	18 (11.9%)	0.58	526 (13.7%)	170 (16.5%)	8 (10.4%)	0.13
Comorbidities									
Diabetes mellitus	334 (6.8%)	134 (5.2%)	164 (7.3%)	36 (23.8%)	< 0.001	226 (5.9%)	81 (7.9%)	27 (35.1%)	< 0.001
Hypertension	923 (18.7%)	318 (12.4%)	520 (23.3%)	85 (56.3%)	< 0.001	631 (16.5%)	253 (24.6%)	39 (50.6%)	< 0.001
Hyperlipidemia	404 (8.2%)	171 (6.7%)	201 (9.0%)	32 (21.2%)	< 0.001	291 (7.6%)	98 (9.5%)	15 (19.5%)	< 0.001
Cardiovascular disease	334 (6.8%)	113 (4.4%)	191 (8.5%)	30 (19.9%)	< 0.001	247 (6.4%)	76 (7.4%)	11 (14.3%)	0.03
Coronary artery disease	50 (1.0%)	14 (0.5%)	32 (1.4%)	4 (2.6%)	< 0.001	40 (1.0%)	8 (0.8%)	2 (2.6%)	0.99
Stroke	39 (0.8%)	14 (0.5%)	23 (1.0%)	2 (1.3%)	0.04	31 (0.8%)	7 (0.7%)	1 (1.3%)	0.91
Laboratory data									
Hemoglobin (g/dL)	14.3 ± 1.5	14.1 ± 1.6	14.6 ± 1.3	14.2 ± 1.8	0.65	14.3 ± 1.5	14.4 ± 1.6	14.5 ± 1.7	0.34
Fasting glucose (mg/dl)	101.2 ± 22.0	99.9 ± 23.0	102.0 ± 20.4	110.4 ± 26.3	< 0.001	99.6 ± 19.3	105.1 ± 27.7	126.8 ± 37.2	< 0.001
BUN (mg/dl)	11.9 ± 3.6	11.0 ± 3.1	12.6 ± 3.4	17.2 ± 5.8	< 0.001	11.7 ± 3.4	12.3 ± 3.9	14.0 ± 5.7	< 0.001
Uric acid (mg/dl)	5.9 ± 1.5	5.5 ± 1.4	6.2 ± 1.4	7.1 ± 1.8	< 0.001	5.9 ± 1.5	5.8 ± 1.5	6.4 ± 1.7	0.003
Creatinine (mg/dl)	0.92 ± 0.20	0.80 ± 0.14	1.02 ± 0.14	1.38 ± 0.24	< 0.001	0.9 ± 0.2	0.9 ± 0.2	1.1 ± 0.3	< 0.001
eGFR (MDRD)	88.5 ± 16.9	100.7 ± 13.0	77.1 ± 7.1	52.4 ± 7.4	< 0.001	89.2 ± 16.5	86.9 ± 17.5	77.2 ± 21.2	< 0.001
eGFR (CKD-EPI)	90.6 ± 15.7	102.9 ± 8.4	79.4 ± 7.6	50.8 ± 7.5	< 0.001	91.4 ± 15.2	88.9 ± 16.6	77.5 ± 21.0	< 0.001
Total cholesterol (mg/dl)	201.6 ± 37.0	198.7 ± 36.1	204.7 ± 35.9	204.2 ± 58.3	0.08	201.4 ± 36.3	201.5 ± 38.7	210.2 ± 46.6	0.04
Triglyceride (mg/dl)	136.2 ± 107.1	130.4 ± 102.8	139.6 ± 78.4	181.9 ± 321.4	< 0.001	133.4 ± 92.8	141.1 ± 145.8	205.2 ± 132.4	< 0.001
LDL (mg/dl)	129.9 ± 33.2	126.8 ± 32.9	133.5 ± 32.7	129.8 ± 38.8	0.29	129.8 ± 33.0	130.1 ± 33.1	133.0 ± 42.7	0.40
HDL (mg/dl)	53.7 ± 15.1	54.8 ± 15.5	52.6 ± 14.7	49.0 ± 12.5	< 0.001	54.0 ± 15.1	52.7 ± 15.1	48.6 ± 14.5	0.002
Albumin (g/dl)	4.5 ± 0.3	4.5 ± 0.3	4.5 ± 0.3	4.4 ± 0.3	< 0.001	4.5 ± 0.3	4.5 ± 0.3	4.4 ± 0.5	< 0.001
Potassium (mEq/l)	4.0 ± 0.3	4.0 ± 0.3	4.0 ± 0.3	4.0 ± 0.4	0.02	4.0 ± 0.3	4.0 ± 0.3	3.9 ± 0.4	0.46
Sodium (mEq/l)	142.2 ± 1.9	142.0 ± 1.8	142.4 ± 1.9	142.0 ± 2.5	0.79	142.2 ± 1.9	142.3 ± 1.9	141.6 ± 2.6	0.03
Chloride (mEq/l)	103.9 ± 2.4	104.0 ± 2.2	103.9 ± 2.4	103.4 ± 3.1	0.01	104.0 ± 2.3	103.7 ± 2.5	102.8 ± 3.4	0.07
Phosphate (mg/dl)	3.6 ± 0.5	3.7 ± 0.5	3.6 ± 0.6	3.4 ± 0.6	< 0.001	3.6 ± 0.5	3.5 ± 0.6	3.5 ± 0.7	0.003
Calcium (mg/dl)	9.3 ± 0.4	9.2 ± 0.4	9.3 ± 0.4	9.4 ± 0.4	< 0.001	9.3 ± 0.4	9.3 ± 0.4	9.3 ± 0.6	0.31
NT-proBNP (pg/ml)	46.9 ± 109.9	35.5 ± 48.5	50.9 ± 100.7	173.5 ± 423.1	< 0.001	43.2 ± 59.3	54.1 ± 175.3	138.7 ± 428.8	< 0.001

eGFR, estimated glomerular filtration rate; BMI, body mass index; SBP, systolic blood pressure; DBP, diastolic blood pressure; BUN, blood urea nitrogen; LDL, low-density lipoprotein; HDL, high-density lipoprotein; NT-proBNP, N-terminal pro-brain natriuretic peptide.

### Echocardiographic findings

On echocardiographic assessment, the systolic function of our participants was preserved (overall LVEF was 62.7 ± 5.4%) (**Table 2**). Overall E/A ratio was 1.2 ± 0.4, E/average e’ 7.9 ± 2.6, septal e’ 8.0 ± 2.2 cm/s, lateral e’ 10.4 ± 2.9 cm/s, average e’ 9.2 ± 2.4 cm/s, LVMI 76.9 ± 14.8 g/m^2^, and NT-proBNP 46.9 ± 109.9 pg/ml. LVMI in our cohort did not meet the criteria for LVH. LV geometry differed significantly by renal function status with higher LVMI, LAV indices, and LV end-diastolic and end-systolic diameters among individuals with lower CKD-EPI eGFR (or higher proteinuria) when compared with their counterparts. In parallel with the severity of renal dysfunction, E/A ratio and e’ gradually decreased, while peak A-wave velocity, DT, IVRT, E/e’, and composite diastolic score all gradually increased (all *p* for trends < 0.05). Similar trends of altered cardiac structures and functions were observed across MDRD eGFR categories (**Supplemental Table 4**). Of note, participants in the worst categories (i.e., having eGFR between 30 and < 60 ml/min/1.73 m^2^ or severe dipstick proteinuria) showed the lowest TDI-determined e’ values (septal e’ < 7 cm/s, lateral e’ < 10 cm/s, and average e’ < 9 cm/s), suggestive of highly abnormal diastolic relaxation (**Table 2**; **Supplemental Table 4**) (16).

**Table 2:** Echocardiographic findings of the entire cohort graded by eGFR and proteinuria.

	All (n = 4942)	CKD-EPI formula			*p* for trend	Proteinuria on Dipstick			*p* for trend
		≥ 90 (n = 2556)	60-89 (n = 2235)	30-59 (n = 151)		None (n = 3835)	Mild (n = 1030)	Severe (n = 77)	
Mitral E (cm/s)	69.2 ± 16.2	71.5 ± 16.2	66.9 ± 15.7	62.9 ± 19.2	< 0.001	69.3 ± 16.1	68.5 ± 16.7	67.9 ± 19.2	0.48
Mitral A (cm/s)	60.8 ± 19.2	57.9 ± 16.6	63.0 ± 20.7	77.0 ± 24.7	< 0.001	60.2 ± 19.2	61.7 ± 18.3	78.4 ± 23.8	< 0.001
E/A ratio	1.2 ± 0.4	1.3 ± 0.4	1.1± 0.4	0.9 ± 0.4	< 0.001	1.2 ± 0.4	1.2 ± 0.5	0.9 ± 0.3	< 0.001
DT (ms)	204.1 ± 39.0	200.4 ± 36.3	207.1 ± 40.5	221.9 ± 50.9	< 0.001	203.6 ± 38.4	204.5 ± 41.1	218.5 ± 42.5	0.002
IVRT (ms)	89.9 ± 15.2	87.9 ± 13.2	91.6 ± 16.1	99.2 ± 23.7	< 0.001	89.6 ± 14.5	90.9 ± 17.2	94.8 ± 18.1	0.01
Septal e’ (cm/s)	8.0 ± 2.2	8.6 ± 2.2	7.5 ± 2.1	5.7 ± 1.7	< 0.001	8.1 ± 2.2	7.7 ± 2.3	6.4 ± 2.3	< 0.001
Lateral e’ (cm/s)	10.4± 2.9	11.1 ± 2.9	9.7 ± 2.7	7.4 ± 2.2	< 0.001	10.5 ± 2.9	10.1 ± 3.0	8.4 ± 2.6	< 0.001
Average e’ (cm/s)	9.2 ± 2.4	9.8 ± 2.4	8.6 ± 2.2	6.6 ± 1.8	< 0.001	9.3 ± 2.4	8.9 ± 2.5	7.4 ± 2.3	< 0.001
E/average e’	7.9 ± 2.6	7.6 ± 2.3	8.2 ± 2.6	10.2 ± 3.9	< 0.001	7.8 ± 2.5	8.1 ± 2.7	9.9 ± 3.4	< 0.001
PAP (mm Hg)	17.1 ± 5.3	16.8 ± 5.0	17.4 ± 5.6	18.3 ± 6.6	0.03	17.1 ± 5.2	16.9 ± 5.4	17.5 ± 5.4	0.63
LVMI (g/m^2^)	76.9 ± 14.8	74.6 ± 14.1	78.7 ± 14.8	86.9 ± 16.1	< 0.001	76.5 ± 14.4	77.8 ± 15.7	86.4 ± 17.7	< 0.001
IVS (mm)	9.0 ± 1.1	8.8 ± 1.1	9.2 ± 1.1	9.8 ± 1.2	< 0.001	9.0 ± 1.1	9.1 ± 1.2	9.6 ± 1.4	< 0.001
LVPW (mm)	9.0 ± 1.1	8.8 ± 1.0	9.2 ± 1.1	9.7 ± 1.0	< 0.001	9.0 ± 1.1	9.1 ± 1.1	9.6 ± 1.3	< 0.001
LVIDd (mm)	46.7 ± 3.6	46.3 ± 3.7	47.1 ± 3.5	48.1 ± 3.5	< 0.001	46.7 ± 3.6	46.7 ± 3.6	48.4 ± 4.1	< 0.001
LVIDs (mm)	29.3 ± 3.0	29.0 ± 2.9	29.5 ± 2.9	30.8 ± 3.9	< 0.001	29.3 ± 2.9	29.2 ± 3.0	30.6 ± 4.0	< 0.001
LVEDV (ml)	76.6 ± 14.3	75.0 ± 14.2	78.0 ± 14.1	82.4 ± 15.5	< 0.001	76.4 ± 14.2	76.7 ± 14.2	83.1 ± 17.2	< 0.001
LVESV (ml)	28.7 ± 7.5	28.0 ± 7.3	29.2 ± 7.3	32.7 ± 12.1	< 0.001	28.6 ± 7.4	28.7 ± 7.8	32.0 ± 11.4	< 0.001
LVEF (%)	62.7 ± 5.4	62.8 ± 5.3	62.7 ± 5.2	60.8 ± 8.2	< 0.001	62.7 ± 5.3	62.7 ± 5.6	61.9 ± 6.7	0.23
SV (ml)	47.9 ± 9.3	47.0 ± 9.2	48.8 ± 9.2	49.7 ± 10.5	0.001	47.8 ± 9.2	48.0 ± 9.4	51.1 ± 10.3	0.002
LAVmax/BSA (ml/m^2^)	16.1 ± 5.8	15.9 ± 5.5	16.3± 6.0	17.4 ± 6.4	0.002	16.1 ± 5.8	16.1 ± 5.7	18.7 ± 7.0	< 0.001
LAVmin/BSA (ml/m^2^)	10.1 ± 7.2	9.7 ± 7.1	10.2 ± 7.3	12.5 ± 8.5	< 0.001	9.9 ± 7.1	10.5 ± 7.5	12.9 ± 8.3	0.001
Composite diastolic score	0.12 ± 0.40	0.10 ± 0.36	0.13 ± 0.41	0.36 ± 0.66	< 0.001	0.11 ± 0.39	0.14 ± 0.43	0.26 ± 0.52	0.001

DT, deceleration time; IVRT, isovolemic relaxation time; PAP, pulmonary artery pressure; LVMI, left ventricular mass index; IVS, interventricular septum thickness; LVPW, left ventricular posterior wall thickness; LVIDd, left ventricular end-diastolic diameter; LVIDs, left ventricular end-systolic diameter; LVEDV, left ventricular end-diastolic volume; LVESV, left ventricular end-systolic volume; LVEF, left ventricular ejection fraction; SV, stroke volume; LAV, left atrial volume; BSA, body surface area.

**Figure 1:**
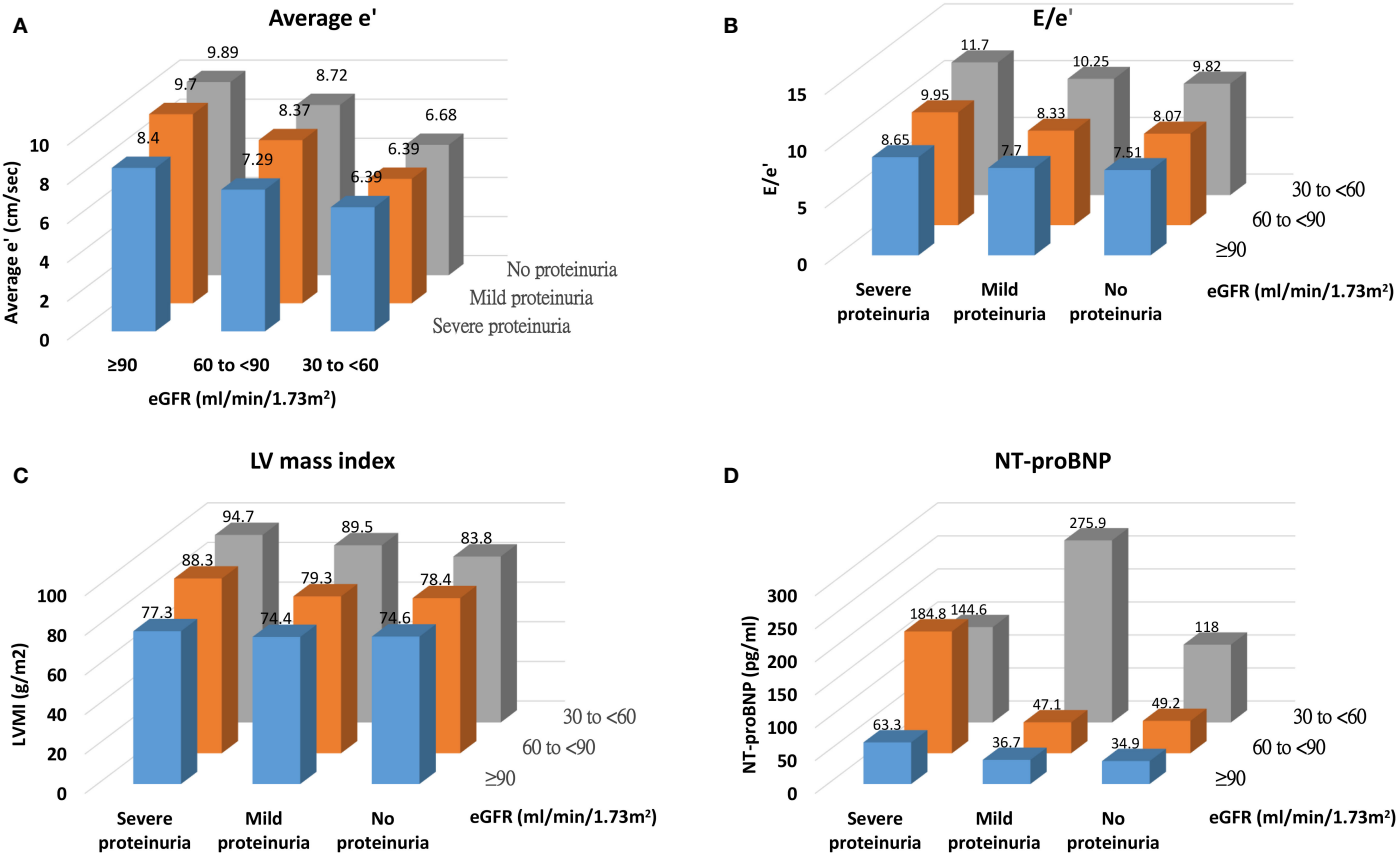
Distribution of cardiac structural and functional parameters [**(A)** average e', **(B)** E/e', **(C)** LV mass index, and **(D)** NT-proBNP] across categories of renal function.

**Figure 1** illustrates the levels of average e’, E/e’, LVMI, and NT-proBNP across categories of eGFR and proteinuria. We demonstrated a graded pattern of average e’, E/e’, and LVMI with the severity of renal function. In **Table 3**, average e’ is summarized by CKD-EPI/MDRD eGFR and proteinuria category. The levels of average e’ did not meet the risk classification for prognosis of CKD and cardiovascular mortality as per the KDIGO guidelines (19).

**Table 3:** Illustrations of average e’ of the entire cohort across graded MDRD/CKD-EPI eGFR and proteinuria categories.

	Average e’ (cm/s)		Proteinuria		
			Normal	Mildly to moderately increased	Severely increased
	CKD-EPI GFR		None	Mild	Severe
1	Normal or high	≥ 90	9.89	9.71	8.40
2	Mildly decreased	60–89	8.72	8.37	7.29
3a	Mildly to moderately decreased	45–59	6.68	6.39	6.39
	Average e’ (cm/sec)		Proteinuria		
			Normal	Mildly to moderately increased	Severely increased
	MDRD GFR		None	Mild	Severe
1	Normal or high	≥ 90	9.82	9.55	8.47
2	Mildly decreased	60–89	8.95	8.63	7.41
3a	Mildly to moderately decreased	45–59	6.80	6.41	6.35

Green, yellow, orange and red cells indicate low, moderately increased, moderate and high relative risks of cardiovascular mortality and prognosis of chronic kidney disease as per the KDIGO 2012 guidelines (19).

### Associations between cardiac diastolic function, renal insufficiency, and circulating NT-proBNP level

In several multivariate regression models adjusted for clinical risk factors, CKD-EPI eGFR was positively correlated with average e’, and negatively correlated with NT-proBNP (**Table 4**) and maximum LAVi (**Supplemental Table 5**). CKD-EPI eGFR had no significant effect on LV filling E/e’ and LVMI. The adjusted models remained significant with respect to markers of DD when CKD-EPI eGFR was replaced by MDRD eGFR (**Table 4**; **Supplemental Table 5**).

**Table 4:** Association of CKD-EPI and MDRD eGFR with markers of diastolic function and cardiac structure in multivariate-adjusted linear regression models.

Variables	CKD-EPI formula							
	Average e’		E/e’		LVMI^†^		NT-proBNP	
(per 10-ml/min/1.73m^2^ increment)	Coef. (95% CI)	*p*-value	Coef. (95% CI)	*p*-value	Coef. (95% CI)	*p*-value	Coef. (95% CI)	*p*-value
Univariate	0.63 (0.59, 0.67)	< 0.001	−0.38 (−0.43, −0.33)	< 0.001	−2.11 (−2.39, −1.83)	< 0.001	−13.6 (−15.6, −11.6)	< 0.001
Multivariate								
Model 1	0.09 (0.05, 0.13)	< 0.001	−0.05 (−0.11, 0.00)	0.06	−0.17 (−0.57, 0.17)	0.32	−12.3 (−14.7, −9.9)	< 0.001
Model 2	0.06 (0.02, 0.10)	0.004	−0.04 (−0.10, 0.01)	0.11	−0.22 (−0.56, 0.11)	0.19	−12.3 (−14.9, −9.8)	< 0.001
Multivariate + Echo Data								
Model 2 + LVMI^†^	0.07 (0.03, 0.11)	0.002	−0.06 (−0.12, 0.00)	0.06	–	–	−12.9 (−15.8, −10.0)	< 0.001
Model 2+ LVEF	0.06 (0.02, 0.10)	0.01	−0.04 (−0.10, 0.01)	0.11	−0.23 (−0.57, 0.11)	0.18	−12.4 (−15.0, −9.8)	< 0.001
Model 2 + SV	0.06 (0.02, 0.10)	0.01	−0.04 (−0.10, 0.01)	0.11	0.12 (−0.17, 0.40)	0.42	−12.6 (−15.2, −10.1)	< 0.001
Multivariate + Echo Data + Proteinuria								
Model 3	0.05 (0.01, 0.09)	0.01	−0.04 (−0.09, 0.02)	0.17	−0.24 (−0.58, 0.10)	0.17	−12.0 (−14.5, −9.4)	< 0.001
Model 3 + LVMI^†^	0.06 (0.02, 0.11)	0.004	−0.05 (−0.11, 0.01)	0.09	–	–	−12.6 (−15.5, −9.7)	< 0.001
Model 3 + LVEF	0.05 (0.01, 0.09)	0.01	−0.04 (−0.09, 0.02)	0.17	−0.25 (−0.58, 0.09)	0.15	−12.1 (−14.6, −9.5)	< 0.001
Model 3 + SV	0.05 (0.01, 0.09)	0.01	−0.04 (−0.09, 0.02)	0.17	0.14 (−0.15, 0.42)	0.35	−12.3 (−14.9, −9.7)	< 0.001
	MDRD formula							
Variables	Average e’		E/e’		LVMI^†^		NT-proBNP	
(per 10-ml/min/1.73m^2^ increment)	Coef. (95% CI)	*p*-value	Coef. (95% CI)	*p*-value	Coef. (95% CI)	*p*-value	Coef. (95% CI)	*p*-value
Univariate	0.40 (0.36, 0.43)	< 0.001	−0.21 (−0.26, −0.17)	< 0.001	−1.16 (−1.42, −0.89)	< 0.001	−9.9 (−11.7, −8.0)	< 0.001
Multivariate								
Model 1	0.08 (0.05, 0.12)	< 0.001	−0.02 (−0.06, 0.02)	0.36	−0.35 (−0.08, −0.63)	0.012	−8.4 (−10.5, −6.4)	< 0.001
Model 2	0.04 (0.01, 0.07)	0.02	−0.03 (−0.07, 0.02)	0.26	−0.36 (−0.09, −0.64)	0.01	−8.5 (−10.6, −6.4)	< 0.001
Multivariate + Echo Data								
Model 2 + LVMI^†^	0.05 (0.01, 0.08)	0.03	−0.03 (−0.08, 0.02)	0.18	–	–	−9.1 (−11.4, −6.7)	< 0.001
Model 2+ LVEF	0.04 (0.01, 0.08)	0.01	−0.03 (−0.07, 0.02)	0.23	−0.37 (−0.09, −0.64)	0.008	−8.6 (−10.7, −6.5)	< 0.001
Model 2 + SV	0.04 (0.01, 0.07)	0.02	−0.03 (−0.07, 0.02)	0.23	0.19 (−0.04, 0.42)	0.11	−8.8 (−10.9, −6.7)	< 0.001
Multivariate + Echo Data + Proteinuria								
Model 3	0.04 (0.00, 0.07)	0.03	−0.02 (−0.06, 0.02)	0.38	−0.38 (−0.10, −0.65)	0.01	−7.9 (−10.0, −5.8)	< 0.001
Model 3 + LVMI^†^	0.04 (0.01, 0.08)	0.02	−0.03 (−0.08, 0.02)	0.26	–	–	−9.5 (−10.8, −6.1)	< 0.001
Model 3 + LVEF	0.04 (0.00, 0.07)	0.03	−0.02 (−0.07, 0.02)	0.36	−0.38 (−0.11, −0.66)	0.01	−8.0 (−10.1, −5.8)	< 0.001
Model 3 + SV	0.04 (0.00, 0.07)	0.04	−0.02 (−0.07, 0.02)	0.35	0.20 (−0.03, 0.43)	0.09	−8.2 (−10.3, −6.1)	< 0.001

Model 1 was adjusted for age + gender;

Model 2 was adjusted for age, gender, BMI, SBP, hypertension, diabetes, CVD, fasting glucose, total cholesterol, HDL, and smoking;

Model 3: Model 2 + proteinuria;

^†^Model 1 and Model 3 were not adjusted for BMI for LVMI.

**Figure 2:**
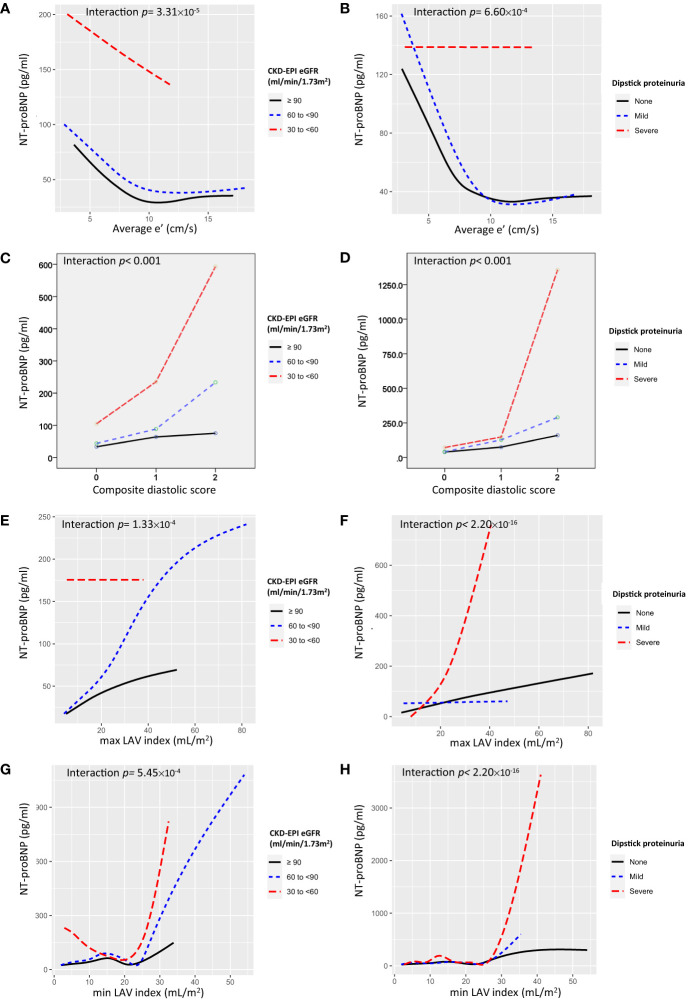
Interaction plots for NT-proBNP for the effects of **(A)** average e’ and eGFR, **(B)** average e’ and proteinuria, **(C)** composite diastolic score and eGFR, **(D)** composite diastolic score and proteinuria, **(E)** maximum LAV index and eGFR, **(F)** maximum LAV index and proteinuria, **(G)** minimum LAV index and eGFR, and **(H)** minimum LAV index and proteinuria.

As shown in **Figure 2**, a significant interaction exists between renal function and diastolic markers with reference to NT-proBNP, (interaction *p* < 0.05). Individuals with lower average e’ or higher composite diastolic score, rather than E/e’, present with higher NT-proBNP levels across worsening eGFR category (or having severe proteinuria) (**Figure 2**; **Supplemental Figure 2**).

## Discussion

This observational study had a large sample size, and describes the associations between renal function and several indices of DD in a cohort without prevalent HF. The majority (97%) of our study participants had a preserved renal function (eGFR of ≥ 60 ml/min/1.73 m^2^). We demonstrated that in the condition of preserved LVEF, lower eGFR, estimated by either by MDRD or CKD-EPI formula, was significantly associated with lower LV e’, greater maximum LAVi, and elevated NT-proBNP, suggesting that abnormal LV structure and diastolic relaxation may be present in subjects with early stages of kidney disease and progress as renal function declines. Instead, E/e’ ratios, markers of LV filling pressures, had a lack of discriminatory power to detect subtle differences in diastolic function in subjects with mild renal impairment. Broadly, average e’ was a more sensitive alternative for the assessment of LV DD in this population.

A noteworthy strength of our study was that we analyzed LV DD with risk stratification by two-dimensional information on GFR and proteinuria. Both markers are pivotal for kidney function, and combined assessment of these two factors is better than either one solely to characterize and to prognosticate CKD progression and relevant morbidities (26). Our study provided objective evidence to demonstrate that eGFR and proteinuria both present independent and synergistic effects on LV structure and DD, even in clinically asymptomatic stages. The pathophysiological mechanism linking renal dysfunction and LV abnormalities has been extensively explored in the past decade. Aside from conventional risk factors such as older age, diabetes, hypertension, smoking, and dyslipidemia (27), some CKD-specific nonconventional factors such as albuminuria (28), LVH (29), fibroblast growth factor 23 (30), deranged mineral metabolism (31), anemia (32) and inflammation (33) may all contribute to CVD. The term cardiorenal syndrome has been increasingly used to describe that severe dysfunction of these organs often occurs in combination rather than in isolation (34). Nevertheless, CKD is a clinical continuum. Our study offered additional insight into heart–kidney interplay, which begins in the early stage of either disease when LVEF and GFR are preserved. To date, early detection of cardiorenal interaction is not easy in the clinically asymptomatic stage without the help of novel biomarkers (such as neutrophil gelatinase-associated lipocalin [NGAL], kidney injury molecule-1 [KIM-1], cystatin C, natriuretic peptides, and cardiac troponins) (35, 36).

On the other hand, NT-proBNP is of the natriuretic peptide family and has excellent *in vitro* stability (37) and diagnostic ability in the assessment of asymptomatic LV dysfunction in patients at risk for HF development (25). NT-proBNP levels are positively correlated with the severity of DD (25, 38); however, interpretation should always consider subjects’ age, gender (25), and renal function (39), and yet data regarding possible interactions between DD and renal function on NT-proBNP level in a large, asymptomatic Asian population remain unexplored. Using NT-proBNP as an indicator of LV DD, our interaction plots showed a marked increase in NT-proBNP in subjects in the severest categories of renal function (i.e., having eGFR between 30 and < 60 ml/min/1.73 m^2^ or heavy dipstick proteinuria) in comparison with those having better renal function. Moreover, an even steeper elevation in NT-proBNP was noted in subjects in the worst renal function categories with parallel lower average e’ (or in the category of composite diastolic score equal to 2 or higher LAV index), rather than E/e’, suggesting that the interaction between heart and kidneys might grow vehemently and disproportionately as either organ begins to lose some function. Of note, although prior studies have reported the utilization of CKD-EPI equation as a more applicable and useful surrogate marker than MDRD for CKD in Asians (40, 41), in our study these two equations displayed similar trends in associations with cardiac diastolic markers.

This study has several limitations. First, although proteinuria or albuminuria is more accurately assessed in terms of urinary protein-to-creatinine or albumin-to-creatinine ratio (ACR), calculated by dividing the urine protein or albumin by urine creatinine during morning urine collection, the urine dipstick test is a simple, fast, and inexpensive tool to screen and diagnose urinary tract problems, including proteinuria. Standard reagent strip dipsticks are especially sensitive to albumin, and even a dipstick test result of trace or higher identifies ACR ≥ 300 mg/g with 100% sensitivity and 83.7% specificity (42). Our study showed a graded pattern of a series of LV measurements with the severity of dipstick results, suggesting that urinalysis is a useful first step to assess proteinuria. Second, the individuals of our cohort were included in a tertiary medical center, which might introduce selection bias. Third, our cohort did not record their drug-taking history. For example, β-blockers, renin–angiotensin–aldosterone system blockers, sodium–glucose cotransporter 2 inhibitors (SGLT2i), and glucagon-like peptide-1 receptor agonists (GLP1 RA) have cardioprotective and renoprotective effects, while non-steroidal anti-inflammatory drugs (NSAIDs) and contrast media may hamper renal function. However, this screening program was conducted between 2009 and 2012, when SGLT2i and GLP1 RA were unavailable. Still, certain missing drug information might elicit treatment bias. Lastly, our database did not contain clinical outcomes, and the correlations to outcomes might be more important than those to surrogate markers.

## Conclusions

In conclusion, in this large cohort of participants with early CKD and without clinical HF, we found a strong association between renal function and LV structural and functional change during diastole. Average e’, instead of E/A or E/e’ ratios, was more sensitive to detect LV DD in this population. Heart–kidney crosstalk starts in the early asymptomatic stage. In this regard, renal function in terms of eGFR and dipstick proteinuria provide crude information on subjects’ LV diastolic function, and prompt interventions might be needed to hinder the devastating cardiorenal crosstalk from the perspective of preventive medicine.

## Data availability statement

The raw data supporting the conclusions of this article will be made available by the authors, without undue reservation.

## Ethics statement

This study was approved by the institutional review board of MacKay Memorial Hospital (14MMHIS202). The informed consent from the patients/participants was waived because of the retrospective nature of this study and the analysis used anonymous clinical data.

## Author contributions

Authors’ contributions: P-CW drafted the manuscript. K-TS, J-LL and T-CH collected data. C-LH and C-JW provided the original conception and design of the study. Y-HL, C-HS and H-IY modified the statistical models critically and provided technical and statistical support during the analyses. All authors contributed to the article and approved the submitted version.
