# Supplementary material for: Relation of early-stage renal insufficiency and cardiac structure and function in a large population of asymptomatic Asians: a cross-sectional cohort analysis
Source: Front Nephrol. 2023 May 12;3:1071900. doi: 10.3389/fneph.2023.1071900 (PMC10479670; doi:10.3389/fneph.2023.1071900)

Supplemental Figure 1: Validation of dipstick proteinuria with quantitative albuminuria in current study.

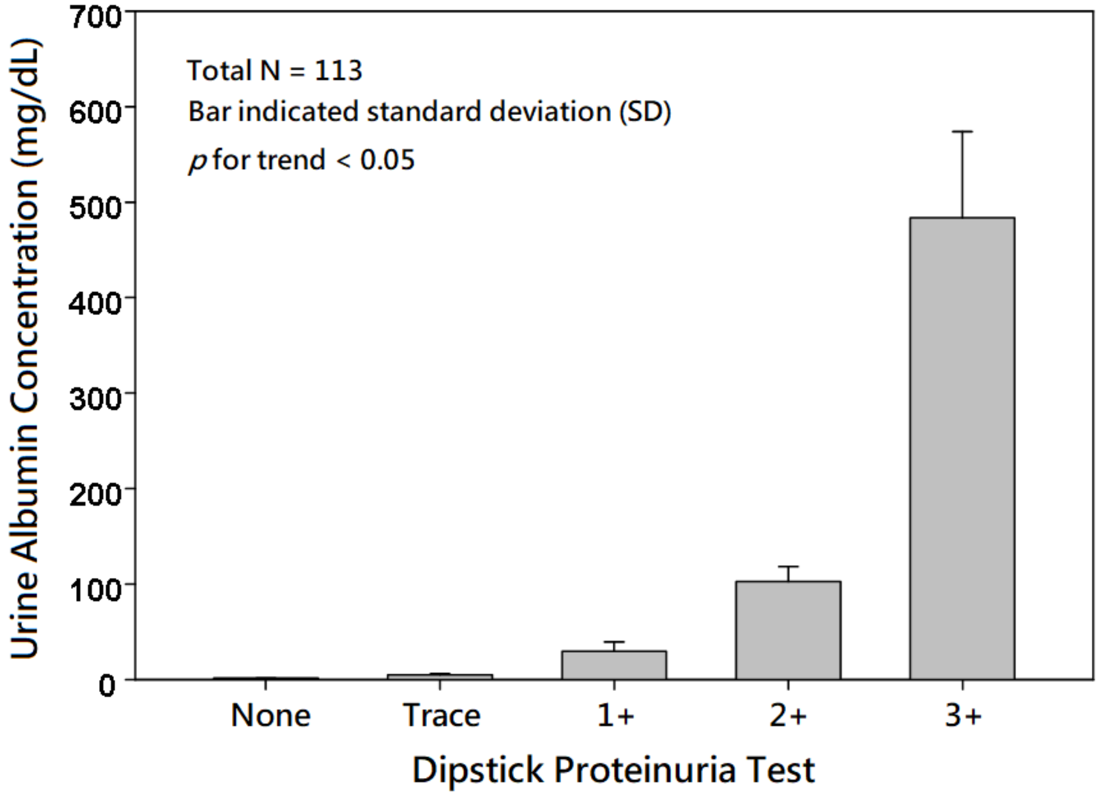

Supplement: Supplementary file 1 [file Image_1.pdf]
