# Supplementary material for: Relation of early-stage renal insufficiency and cardiac structure and function in a large population of asymptomatic Asians: a cross-sectional cohort analysis
Source: Front Nephrol. 2023 May 12;3:1071900. doi: 10.3389/fneph.2023.1071900 (PMC10479670; doi:10.3389/fneph.2023.1071900)
Supplement: Supplementary file 2 [file Image_2.pdf]

Supplemental Figure 2: Interaction plots for NT-proBNP for the effects of (A) quartiles of average e' and MDRD eGFR, and (B) composite diastolic score and MDRD eGFR.

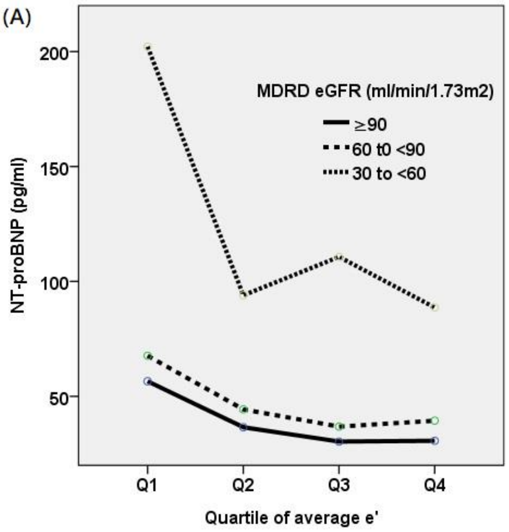

Variable	Interaction *p*	Partial Eta Squared
Quartiles of average e'	<0.001	0.011
eGFR categories	<0.001	0.006
Average e' x eGFR	0.013	0.003

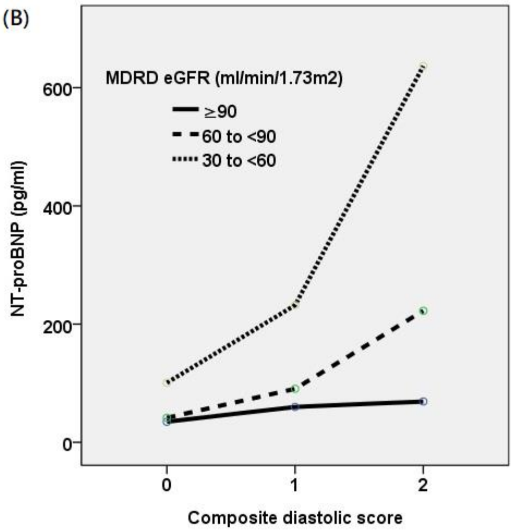

Variable	Interaction *p*	Partial Eta Squared
Composite diastolic score	<0.001	0.080
eGFR categories	<0.001	0.071
Score x eGFR	<0.001	0.042
