# Supplementary material for: Relation of early-stage renal insufficiency and cardiac structure and function in a large population of asymptomatic Asians: a cross-sectional cohort analysis
Source: Front Nephrol. 2023 May 12;3:1071900. doi: 10.3389/fneph.2023.1071900 (PMC10479670; doi:10.3389/fneph.2023.1071900)
Supplement: Supplementary file 3 [file Table_1.docx]

**Supplemental Table 1**: Reproducibility analysis of echocardiographic measurements

| **Coefficients of variance (%)** | **LVEDV (mL)** | **LAVmax (mL)** |
| --- | --- | --- |
| Interobserver variability | 7.6 | 7.1 |
| Intraobserver variability | 5.9 | 6.2 |

Abbreviations: GLS, global longitudinal strain; LVEDV, left ventricular end-diastolic volume; LAVmax, maximal left atrial volume.
