# Supplementary material for: Relation of early-stage renal insufficiency and cardiac structure and function in a large population of asymptomatic Asians: a cross-sectional cohort analysis
Source: Front Nephrol. 2023 May 12;3:1071900. doi: 10.3389/fneph.2023.1071900 (PMC10479670; doi:10.3389/fneph.2023.1071900)
Supplement: Supplementary file 4 [file Table_2.docx]

**Supplemental Table 2**: Clinical characteristics between participants who were included versus excluded in the current analysis

|  | **Included**  **(n = 4942)** | **Excluded**  **(n = 584)** | ***p-value*** |
| --- | --- | --- | --- |
|  |  |  |  |
| **Patient characteristics** |  |  |  |
| Age (year) | 49.4 ± 11.2 | 51.7 ± 12.7 | <0.001 |
| Male gender | 3254 (65.8%) | 303 (51.9%) | <0.001 |
| Height (cm) | 165.6 ± 8.5 | 163.4 ± 9.3 | <0.001 |
| Weight (kg) | 67.4 ± 12.9 | 65.3 ± 12.6 | 0.001 |
| BMI (kg/cm^2^) | 24.4 ± 3.6 | 24.3 ± 3.7 | 0.53 |
| Body fat (%) | 26.2 ± 6.7 | 13.3 ± 14.3 | <0.001 |
| SBP (mm Hg) | 122.9 ± 17.2 | 125.6 ± 19.4 | <0.001 |
| DBP (mm Hg) | 75.8 ± 10.9 | 73.6 ± 11.9 | <0.001 |
| Pulse rate (/min) | 74.4 ± 10.2 | 74.0 ± 11.5 | 0.38 |
| Current smoking | 543 (11.0%) | 29 (5.0%) | <0.001 |
| Exercise | 704 (14.2%) | 41 (7.0%) | <0.001 |
| **Comorbidities** |  |  |  |
| Diabetes mellitus | 334 (6.8%) | 64 (11.0%) | <0.001 |
| Hypertension | 923 (18.7%) | 115 (19.7%) | 0.55 |
| Hyperlipidemia | 404 (8.2%) | 93 (15.9%) | <0.001 |
| Cardiovascular disease | 334 (6.8%) | 70 (12.0%) | <0.001 |
| Coronary artery disease | 0 (0%) | 29 (5.0%) | <0.001 |
| Stroke | 39 (0.8%) | 9 (1.5%) | 0.06 |
| **Laboratory data** |  |  |  |
| Hemoglobin (g/dL) | 14.3 ± 1.5 | 13.8 ± 1.7 | <0.001 |
| Fasting glucose (mg/dl) | 101.2 ± 22.0 | 100.4 ± 20.3 | 0.40 |
| BUN (mg/dl) | 11.9 ± 3.6 | 12.8 ± 6.1 | <0.001 |
| Uric acid (mg/dl) | 5.9 ± 1.5 | 5.8 ± 1.5 | 0.13 |
| Creatinine (mg/dl) | 0.92 ± 0.20 | 0.98 ± 0.81 | <0.001 |
| eGFR (CKD-EPI) | 90.6 ± 15.7 | 90.2 ± 19.0 | 0.57 |
| eGFR (MDRD) | 88.5 ± 17.1 | 88.1 ± 19.7 | 0.74 |
| Total cholesterol (mg/dl) | 201.6 ± 37.0 | 202.7 ± 35.0 | 0.49 |
| Triglyceride (mg/dl) | 136.2 ± 107.1 | 121.2 ± 79.9 | 0.001 |
| LDL (mg/dl) | 129.9 ± 33.2 | 130.4 ± 32.5 | 0.73 |
| HDL (mg/dl) | 53.7 ± 15.1 | 56.8 ± 14.9 | <0.001 |
| Albumin (g/dl) | 4.5 ± 0.3 | 4.5 ± 0.4 | 0.31 |
| Potassium (mEq/l) | 4.0 ± 0.3 | 4.0 ± 0.3 | 0.11 |
| Sodium (mEq/l) | 142.2 ± 1.9 | 140.4 ± 3.3 | <0.001 |
| Chloride (mEq/l) | 103.9 ± 2.4 | 103.6 ± 2.1 | 0.004 |
| Phosphate (mg/dl) | 3.6 ± 0.5 | 3.9 ± 0.5 | <0.001 |
| Calcium (mg/dl) | 9.3 ± 0.4 | 9.0 ± 0.5 | <0.001 |

Abbreviations: BMI, body mass index; SBP, systolic blood pressure; DBP, diastolic blood pressure; BUN, blood urea nitrogen; eGFR, estimated glomerular filtration rate; LDL, low-density lipoprotein; HDL, high-density lipoprotein.
