# Supplementary material for: Relation of early-stage renal insufficiency and cardiac structure and function in a large population of asymptomatic Asians: a cross-sectional cohort analysis
Source: Front Nephrol. 2023 May 12;3:1071900. doi: 10.3389/fneph.2023.1071900 (PMC10479670; doi:10.3389/fneph.2023.1071900)
Supplement: Supplementary file 5 [file Table_3.docx]

**Supplemental Table 3**: Echocardiographic findings between participants who were included versus excluded in the current analysis

|  | **Included**  **(n = 4942)** | **Excluded**  **(n = 584)** | ***p*-value** |
| --- | --- | --- | --- |
|  |  |  |  |
| **LV mass** |  |  |  |
| LVMi (g/m^2^) | 76.9 ± 14.8 | 76.3 ± 18.1 | 0.46 |
| IVS (mm) | 9.0 ± 1.1 | 9.0 ± 1.3 | 0.89 |
| LVPW (mm) | 9.0 ± 1.1 | 9.1 ± 1.2 | 0.46 |
| **LV size** |  |  |  |
| LVEDD (mm) | 46.7 ± 3.6 | 45.9 ± 4.0 | <0.001 |
| LVESD (mm) | 29.3 ± 3.0 | 29.1 ± 3.6 | 0.14 |
| LVEDV (ml) | 76.6 ± 14.3 | 73.7 ± 15.2 | <0.001 |
| LVESV (ml) | 28.7 ± 7.5 | 28.3 ± 8.0 | 0.23 |
| **LV systolic function** |  |  |  |
| LVEF (%) | 62.7 ± 5.4 | 61.9 ± 6.4 | <0.001 |
| GLS (%) | -20.1 ± 1.9 | -19.9 ± 2.0 | <0.001 |
| **LV diastolic function** |  |  |  |
| E/A ratio | 1.2 ± 0.4 | 1.2 ± 0.4 | 0.01 |
| DT (ms) | 204.1 ± 39.0 | 207.4 ± 42.4 | 0.06 |
| IVRT (ms) | 89.9 ± 15.2 | 86.0 ± 16.1 | <0.001 |
| Septal e’ (cm/s) | 8.0 ± 2.2 | 7.8 ± 2.3 | 0.04 |
| Lateral e’ (cm/s) | 10.4 ± 2.9 | 10.0 ± 3.1 | 0.002 |
| Average e’ (cm/s) | 9.2 ± 2.4 | 8.9 ± 2.5 | 0.19 |
| Average E/e’ | 7.9 ± 2.6 | 8.2 ± 2.8 | 0.01 |
| max LAVi (ml/m^2^) | 16.1 ± 5.8 | 16.0 ± 6.0 | 0.69 |
| min LAVi (ml/m^2^) | 10.1 ± 7.2 | 10.3 ± 7.7 | 0.53 |
| Composite diastolic score | 0.12 ± 0.40 | 0.13 ± 0.34 | 0.56 |
| **Other** |  |  |  |
| NT-proBNP (pg/ml) | 46.9 ± 109.9 | 45.0 ± 60.3 | 0.68 |

Abbreviations: LVMi, left ventricular mass index; IVS, interventricular septum thickness; LVPW, left ventricular posterior wall thickness; LVEDD, left ventricular end-diastolic diameter; LVESD, left ventricular end-systolic diameter; LVEDV, left ventricular end-diastolic volume; LVESV, left ventricular end-systolic volume; LVEF, left ventricular ejection fraction; GLS, global longitudinal strain; DT, deceleration time; IVRT, isovolemic relaxation time; LAVi, left atrial volume index; NT-proBNP, N-terminal pro-brain natriuretic peptide
