# Supplementary material for: Relation of early-stage renal insufficiency and cardiac structure and function in a large population of asymptomatic Asians: a cross-sectional cohort analysis
Source: Front Nephrol. 2023 May 12;3:1071900. doi: 10.3389/fneph.2023.1071900 (PMC10479670; doi:10.3389/fneph.2023.1071900)
Supplement: Supplementary file 6 [file Table_4.docx]

**Supplemental Table 4**: Echocardiographic findings and NT-proBNP levels graded by MDRD eGFR

|  | **All**  **(n = 4942)** | **MDRD eGFR** | | | ***p* for trend** | **Adjusted *p*** | | |
| --- | --- | --- | --- | --- | --- | --- | --- | --- |
|  |  | **≥ 90** | **60–89** | **30–59** |  | **Model 1** | **Model 2** | **Model 3** |
|  |  | **(n = 2556)** | **(n = 2235)** | **(n = 151)** |  |  |  |  |
| **LV mass** |  |  |  |  |  |  |  |  |
| LVMi (g/m^2^) | 76.9 ± 14.8 | 75.2 ± 14.4 | 77.7 ± 14.6 | 87.0 ± 16.3 | <0.001 | 0.01 | 0.01 | 0.01 |
| IVS (mm) | 9.0 ± 1.1 | 8.9 ± 1.1 | 9.1 ± 1.1 | 9.8 ± 1.2 | <0.001 | 0.16 | 0.60 | 0.65 |
| LVPW (mm) | 9.0 ± 1.1 | 8.9 ± 1.0 | 9.1 ± 1.1 | 9.7 ± 1.0 | <0.001 | 0.21 | 0.71 | 0.74 |
| **LV size** |  |  |  |  |  |  |  |  |
| LVEDD (mm) | 46.7 ± 3.6 | 46.3 ± 3.7 | 47.1 ± 3.5 | 48.1 ± 3.5 | <0.001 | 0.33 | 0.07 | 0.08 |
| LVESD (mm) | 29.3 ± 3.0 | 29.0 ± 3.0 | 29.5 ± 2.8 | 30.6 ± 3.9 | <0.001 | 0.97 | 0.56 | 0.59 |
| LVEDV (ml) | 76.6 ± 14.3 | 74.7 ± 14.3 | 77.8 ± 14.0 | 81.8 ± 14.7 | <0.001 | 0.44 | 0.16 | 0.15 |
| LVESV (ml) | 28.7 ± 7.5 | 27.9 ± 7.4 | 29.2 ± 7.3 | 32.2 ± 12.0 | <0.001 | 0.75 | 0.94 | 0.94 |
| **LV systolic function** |  |  |  |  |  |  |  |  |
| LVEF (%) | 62.7 ± 5.4 | 62.9 ± 5.4 | 62.7 ± 5.2 | 61.1 ± 8.3 | <0.001 | 0.31 | 0.29 | 0.28 |
| GLS (%) | -20.1 ± 1.9 | -20.4 ± 1.9 | -19.9 ± 1.8 | -18.7 ± 2.4 | <0.001 | 0.05 | 0.11 | 0.16 |
| **LV diastolic function** |  |  |  |  |  |  |  |  |
| E/A ratio | 1.2 ± 0.4 | 1.3 ± 0.4 | 1.2 ± 0.4 | 0.9 ± 0.5 | <0.001 | 0.31 | 0.93 | 0.84 |
| DT (ms) | 204.1 ± 39.0 | 200.6 ± 36.6 | 206.0 ± 39.9 | 220.8 ± 51.4 | <0.001 | 0.02 | 0.04 | 0.04 |
| IVRT (ms) | 89.9 ± 15.2 | 88.0 ± 13.5 | 91.1 ± 15.6 | 98.4 ± 23.8 | <0.001 | 0.01 | 0.02 | 0.03 |
| Septal e’ (cm/s) | 8.0 ± 2.2 | 8.5 ± 2.3 | 7.7 ± 2.1 | 5.8 ± 1.7 | <0.001 | 0.03 | 0.16 | 0.27 |
| Lateral e’ (cm/s) | 10.4 ± 2.9 | 11.0 ± 3.0 | 10.0 ± 2.7 | 7.5 ± 2.2 | <0.001 | 0.002 | 0.01 | 0.01 |
| Average e’ (cm/s) | 9.2 ± 2.4 | 9.8 ± 2.4 | 8.9 ± 2.3 | 6.6 ± 1.8 | <0.001 | 0.003 | 0.02 | 0.03 |
| Average E/e’ | 7.9 ± 2.6 | 7.6 ± 2.3 | 8.0 ± 2.6 | 10.0 ± 4.0 | <0.001 | 0.36 | 0.26 | 0.38 |
| max LAVi (ml/m^2^) | 16.1 ± 5.8 | 16.0 ± 5.6 | 16.2 ± 5.9 | 17.3 ± 6.5 | 0.01 | 0.004 | 0.002 | 0.002 |
| min LAVi (ml/m^2^) | 10.1 ± 7.2 | 9.9 ± 7.2 | 10.0 ± 7.2 | 12.6 ± 8.6 | <0.001 | 0.29 | 0.39 | 0.52 |
| Composite diastolic score | 0.12 ± 0.40 | 0.10 ± 0.36 | 0.12 ± 0.40 | 0.36 ± 0.65 | <0.001 | **–** | **–** | **–** |
| **Other** |  |  |  |  |  |  |  |  |
| NT-proBNP (pg/ml) | 46.9 ± 109.9 | 36.5 ± 53.2 | 48.2 ± 93.7 | 172.5 ± 431.0 | <0.001 | <0.001 | <0.001 | <0.001 |

Abbreviations: NT-proBNP, N-terminal pro-brain natriuretic peptide; LVMi, left ventricular mass index; IVS, interventricular septum thickness; LVPW, left ventricular posterior wall thickness; LVEDD, left ventricular end-diastolic diameter; LVESD, left ventricular end-systolic diameter; LVEDV, left ventricular end-diastolic volume; LVESV, left ventricular end-systolic volume; LVEF, left ventricular ejection fraction; GLS, global longitudinal strain; DT, deceleration time; IVRT, isovolemic relaxation time; LAVi, left atrial volume index.

Model 1 was adjusted for age + gender;

Model 2 was adjusted for age, gender, BMI, SBP, hypertension, diabetes, CVD, fasting glucose, total cholesterol, HDL, and smoking;

Model 3: Model 2 + proteinuria.

* BMI was not incorporated in the adjusted models for LVMi and LAVi
