# Supplementary material for: Relation of early-stage renal insufficiency and cardiac structure and function in a large population of asymptomatic Asians: a cross-sectional cohort analysis
Source: Front Nephrol. 2023 May 12;3:1071900. doi: 10.3389/fneph.2023.1071900 (PMC10479670; doi:10.3389/fneph.2023.1071900)
Supplement: Supplementary file 7 [file Table_5.docx]

**Supplemental Table 5**: Association of CKD-EPI/MDRD eGFR and proteinuria with LAV index in multivariate-adjusted linear regression models

| **eGFR** (per 10-ml/min/1.73m^2^ increment) | **CKD-EPI** | | | | **MDRD** | | | |
| --- | --- | --- | --- | --- | --- | --- | --- | --- |
|  | **Maximum LAVi** | | **Minimum LAVi** | | **Maximum LAVi** | | **Minimum LAVi** | |
|  | Coef. (95% CI) | *p*-value | Coef. (95% CI) | *p*-value | Coef. (95% CI) | *p*-value | Coef. (95% CI) | *p*-value |
| **Univariate** | −0.38 (−0.49, −0.27) | <0.001 | −0.36 (−0.49, −0.22) | <0.001 | −0.13 (−0.23, −0.02) | 0.02 | −0.18 (−0.31, −0.05) | 0.01 |
| **Multivariate** |  |  |  |  |  |  |  |  |
| Model 1 | 0.10 (−0.04, 0.23) | 0.15 | 0.15 (−0.32, 0.02) | 0.07 | −0.16 (−0.05, −0.27) | 0.004 | −0.07 (−0.21, 0.06) | 0.29 |
| Model 2 | −0.16 (−0.02, −0.29) | 0.02 | −0.13 (−0.30, 0.04) | 0.14 | −0.17 (−0.06, −0.28) | 0.002 | −0.07 (−0.21, 0.07) | 0.33 |
| **Multivariate + Echo Data** |  |  |  |  |  |  |  |  |
| Model 2 + LVMi | −0.22 (−0.08, −0.36) | 0.002 | −0.14 (−0.33, 0.06) | 0.16 | −0.22 (−0.11, −0.34) | <0.001 | −0.09 (−0.25, 0.07) | 0.27 |
| Model 2+ LVEF | −0.16 (−0.02, −0.29) | 0.02 | −0.13 (−0.30, 0.04) | 0.13 | −0.17 (−0.06, −0.29) | 0.002 | −0.07 (−0.21, 0.07) | 0.32 |
| Model 2 + SV | −0.15 (−0.01, −0.28) | 0.03 | −0.13 (−0.30, 0.04) | 0.13 | −0.16 (−0.05, −0.27) | 0.004 | −0.08 (−0.22, 0.06) | 0.27 |
| **Multivariate + Echo Data + Proteinuria** |  |  |  |  |  |  |  |  |
| Model 3 | −0.16 (−0.03, −0.29) | 0.02 | −0.11 (−0.28, 0.06) | 0.22 | −0.17 (−0.06, −0.28) | 0.002 | −0.05 (−0.19, 0.09) | 0.46 |
| Model 3 + LVMi | −0.21 (−0.07, −0.36) | 0.003 | −0.11 (−0.30, 0.08) | 0.26 | −0.22 (−0.11, −0.34) | <0.001 | −0.07 (−0.23, 0.09) | 0.39 |
| Model 3 + LVEF | −0.16 (−0.03, −0.29) | 0.02 | −0.11 (−0.28, 0.06) | 0.21 | −0.17 (−0.06, −0.29) | 0.002 | −0.06 (−0.20, 0.09) | 0.44 |
| Model 3 + SV | −0.15 (−0.02, −0.28) | 0.03 | −0.11 (−0.28, 0.06) | 0.20 | −0.16 (−0.05, −0.27) | 0.004 | −0.06 (−0.20, 0.08) | 0.38 |

| **Proteinuria** | **Maximum LAVi** | | | | **Minimum LAVi** | | | |
| --- | --- | --- | --- | --- | --- | --- | --- | --- |
|  | Coef. (95% CI) | | *p*-value | | Coef. (95% CI) | | *p*-value | |
| **Univariate** | 0.28 (−0.13, 0.69) | | 0.18 | | 0.86 (0.40, 1.31) | | <0.001 | |
| **Multivariate** |  | |  | |  | |  | |
| Model 1 | 0.17 (−0.23, 0.57) | | 0.41 | | 0.77 (0.31, 1.22) | | 0.001 | |
| Model 2 | −0.04 (−0.45, 0.36) | | 0.83 | | 0.62 (0.16, 1.08) | | 0.01 | |
| **Multivariate + Echo Data** |  | |  | |  | |  | |
| Model 2 + LVMi | −0.23 (−0.66, 0.20) | | 0.30 | | 0.81 (0.28, 1.33) | | 0.003 | |
| Model 2+ LVEF | −0.02 (−0.43, 0.38) | | 0.91 | | 0.01 | | 0.01 | |
| Model 2 + SV | −0.01 (−0.41. 0.38) | | 0.95 | | 0.01 | | 0.01 | |
|  | **CKD-EPI** | | **MDRD** | | **CKD-EPI** | | **MDRD** | |
|  | Coef. (95% CI) | *p*-value | Coef. (95% CI) | *p*-value | Coef. (95% CI) | *p*-value | Coef. (95% CI) | *p*-value |
| **Multivariate + Echo Data + eGFR** |  |  |  |  |  |  |  |  |
| Model 4 | −0.01 (−0.42, 0.39) | 0.95 | 0.01 (−0.40, 0.41) | 0.99 | 0.60 (0.13, 1.06) | 0.01 | 0.56 (0.05, 1.08) | 0.03 |
| Model 4 + LVMi | −0.19 (−0.62, 0.25) | 0.40 | −0.18 (−0.61, 0.26) | 0.43 | 0.78 (0.26, 1.31) | 0.004 | 0.77 (0.19, 1.36) | 0.01 |
| Model 4 + LVEF | 0.01 (−0.40, 0.42) | 0.97 | 0.03 (−0.02, 0.04) | 0.91 | 0.61 (0.14, 1.07) | 0.01 | 0.57 (0.06, 1.08) | 0.03 |
| Model 4 + SV | 0.02 (−0.38, 0.41) | 0.93 | 0.03 (−0.37, 0.43) | 0.88 | 0.60 (0.13, 1.06) | 0.01 | 0.58 (0.06, 1.09) | 0.03 |

Model 1 was adjusted for age + gender;

Model 2 was adjusted for age, gender, SBP, hypertension, diabetes, CVD, fasting glucose, total cholesterol, HDL, and smoking;

Model 3: Model 2 + proteinuria;

Model 4: Model 2 + eGFR
